# Neutrophil-Predominant Peritoneal Tuberculosis With Salpingitis Mimicking Ovarian Cancer: A Case Report

**DOI:** 10.7759/cureus.88009

**Published:** 2025-07-15

**Authors:** Noura Almarzooqi, Radwa Kafafi, Aymen Abbas, Amal Alghefari

**Affiliations:** 1 Internal Medicine, Sheikh Khalifa Medical City, Abu Dhabi, ARE

**Keywords:** abdominal, cancer, extrapulmonary, genital, malignancy, ovarian, peritonitis, salpingitis, salpinx, tuberculosis

## Abstract

Tuberculosis (TB) is caused by *Mycobacterium tuberculosis*. It is a multisystem infection, but the most common manifestation is pulmonary. TB is the leading cause of death among infectious diseases. Abdominal TB is a form of extrapulmonary TB (EPTB) that often presents nonspecifically and can result from genitourinary or hematogenous spread. We present the case of a 20-year-old Bangladeshi woman who presented with a two-month history of fever and night sweats accompanied by vomiting, tachycardia, and tachypnea. Laboratory investigations revealed elevated inflammatory markers, lactate dehydrogenase, and cancer antigen 125. QuantiFERON-TB (QIAGEN N.V., Venlo, Netherlands) was positive. An abdominal ultrasound showed free fluid throughout the abdomen. A CT scan of the abdomen revealed thickening and enhancement of the peritoneum, omental caking, and bilateral bulky ovaries measuring up to 8.5 x 5.4 cm on the right side, raising suspicion of malignancy. However, an MRI showed that the pelvic masses were, in fact, bilateral hydrosalpinx. Peritoneal analysis indicated a serum ascites albumin gradient (SAAG) of less than 1.1, suggesting peritonitis with neutrophil predominance. Acid-fast bacillus (AFB) culture of the peritoneal fluid grew *M. tuberculosis*. Abdominal TB and ovarian cancers can overlap in clinical presentation, especially in young female patients. Imaging may be misleading, as seen in this case. Imaging modalities may assist in the diagnosis; further exploration, particularly diagnostic laparoscopy with biopsy, is required for suspected ovarian cancer and EPTB.

## Introduction

Tuberculosis (TB) is caused by *Mycobacterium tuberculosis* and usually manifests as a pulmonary infection, but it can affect any organ. Extrapulmonary TB (EPTB) occurs in 10-42% of patients, with or without pulmonary involvement [1]. TB is preventable and treatable, yet it causes 1.5 million deaths annually, making it the leading infectious disease killer. In 2023, TB incidence rate was 134 new cases per 100,000 population, predominantly in 30 high-burden countries, which accounted for 87% of the global cases; Five countries accounted for 56% of worldwide cases: India (26%), Indonesia (10%), China (6.8%), the Philippines (6.8%), and Pakistan (6.3%) [2]. Major risk factors include poverty, overcrowding, inadequate healthcare, poor ventilation, diabetes, substance abuse, kidney disease, and HIV [3].

Abdominal TB is a notable EPTB, affecting 1-2% of all TB cases and involving the intestine, liver, spleen, female genital tract, and peritoneum. It typically reactivates dormant infections but can arise from genitourinary sites or hematogenous spread from pulmonary lesions. Due to its nonspecific symptoms, such as abdominal distension, ascites, tenderness, fever, and weight loss, diagnostic delays can occur. A high level of suspicion is essential for the diagnosis of abdominal TB, especially in individuals aged 20-40 years, unlike genital cancer, which usually affects older populations [4]. We present a case of peritoneal TB with genital involvement characterized by salpingitis, which was initially misidentified as a malignancy. Its uniqueness lies in the peritoneal fluid being unusually predominant in neutrophils.

## Case presentation

A 20-year-old Bangladeshi female patient with no known comorbidities presented with dizziness and collapsed without losing consciousness after experiencing sudden chest pain, abdominal pain, and bilateral leg pain. She reported a two-month history of fever and night sweats associated with occasional non-bloody vomiting and a productive cough of yellowish sputum. A review of systems was unremarkable, including no history of weight loss, bowel or urinary changes, and vaginal discharge. She denied any exposure to sick contacts, family history of any diseases, history of smoking, illicit drug use, recent travel, exposure to animals, or consumption of unpasteurized dairy products. She drank alcohol occasionally and was sexually active with a single partner without any methods of protection. The patient was not monitoring her menstrual cycles. 

The patient was febrile at 38 °C, with sinus tachycardia and tachypnoea and a normal blood pressure of 123/79 mmHg. Her chest was clear upon auscultation, with normal heart sounds and no murmurs detected. Her abdomen was soft but distended, showing mild tenderness in the lower quadrants without guarding or rigidity. Skin exhibited multiple scratch marks extending from the forearms to the elbows. No signs of rashes were observed. There was no lymphadenopathy. The gynecological examination was unremarkable; the vaginal canal and cervix showed no discharges or erythematous lesions, and no ulcers were present. There was no tenderness with cervical motion.

Laboratory investigations revealed deranged liver enzymes with an aspartate aminotransferase (AST):alanine transaminase (ALT) ratio of >2:1, raised cancer antigen 125 (CA125), raised lactate dehydrogenase (LDH), raised C-reactive protein (CRP), hypoalbuminemia, iron deficiency anemia, and mild hyponatremia (Table 1). QuantiFERON-TB (QIAGEN N.V., Venlo, Netherlands) was positive. Other investigations included viral markers for human immunodeficiency virus and hepatitis A, B, C, and E, screening for syphilis, malaria, and autoimmune panels, which were all negative. A high vaginal swab was positive for bacterial vaginosis. The chest X-ray was unremarkable, showing no consolidations or pleural effusion, with a normal heart silhouette.

**Table 1:** Laboratory test results MCV: mean corpuscular volume; AST: aspartate aminotransferase; ALT: alanine transaminase; LDH: lactate dehydrogenase; CA125: cancer antigen 125

Laboratory test	Reference Range	Patient Value
Hemoglobin (g/dL)	11.9-14.8	10.5
MCV (fL)	82.5-98	77.2
Iron (microM/L)	10.7-26.7	3.3
Transferrin (g/L)	2.00-3.50	0.90
Ferritin (mcg/L)	40-200	2,003
Sodium (mmol/L)	136-145	128
C‐reactive protein (mg/dl)	≤0.8	81.7
AST (IU/L)	10-40	106
ALT (IU/L)	10-40	46
Albumin (g/L)	35-55	25
LDH (units/L)	80-225	650
CA125 (units/mL)	<35	142

**Figure 1:**
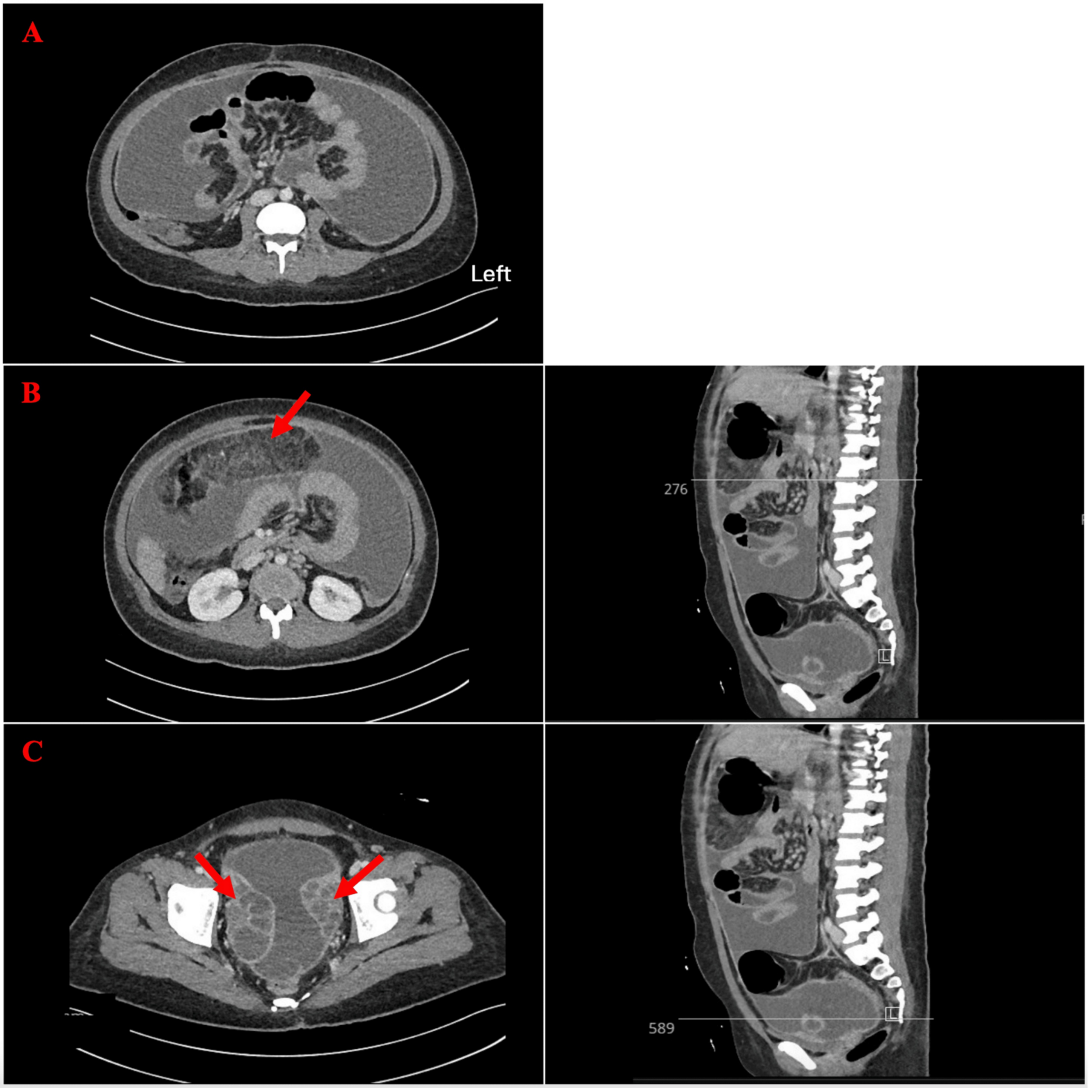
Abdomen and pelvis CT with contrast (a) Transverse section of the abdomen showing a significant volume of abdominopelvic free fluid collection noted in the mid-abdomen, accompanied by clustered bowel; (b) Transverse and sagittal sections of the abdomen showing omental fat stranding and nodularity anterior to the transverse colon, suggestive of omental caking (arrow); (c) Transverse and sagittal sections of the pelvis showing, at the acetabulum level, bilateral bulky ovaries measuring approximately 8.5 x 5.4 cm on the right side with multiple large follicles and 6 x 4.5 cm on the left side with multiple follicles (arrows)

The abdominal ultrasound reported an estimated 2000 ml of ascitic fluid detected all over the abdomen and pelvis, with some internal thick septations noted at the left upper quadrant region. A subsequent computed tomography (CT) of the abdomen and pelvis with contrast revealed a significant volume of free abdominopelvic fluid, with thickening and enhancement of the peritoneum, exhibiting features suggestive of peritonitis. No apparent peritoneal nodules or mass lesions were visualized. There was omental fat stranding and nodularity anterior to the transverse colon, indicative of omental caking. The bilateral bulky ovaries measured approximately 8.5 x 5.4 cm on the right side, displaying multiple large follicles or cysts, and 6 x 4.5 cm on the left side, containing multiple follicles (Figure 1).

**Figure 2:**
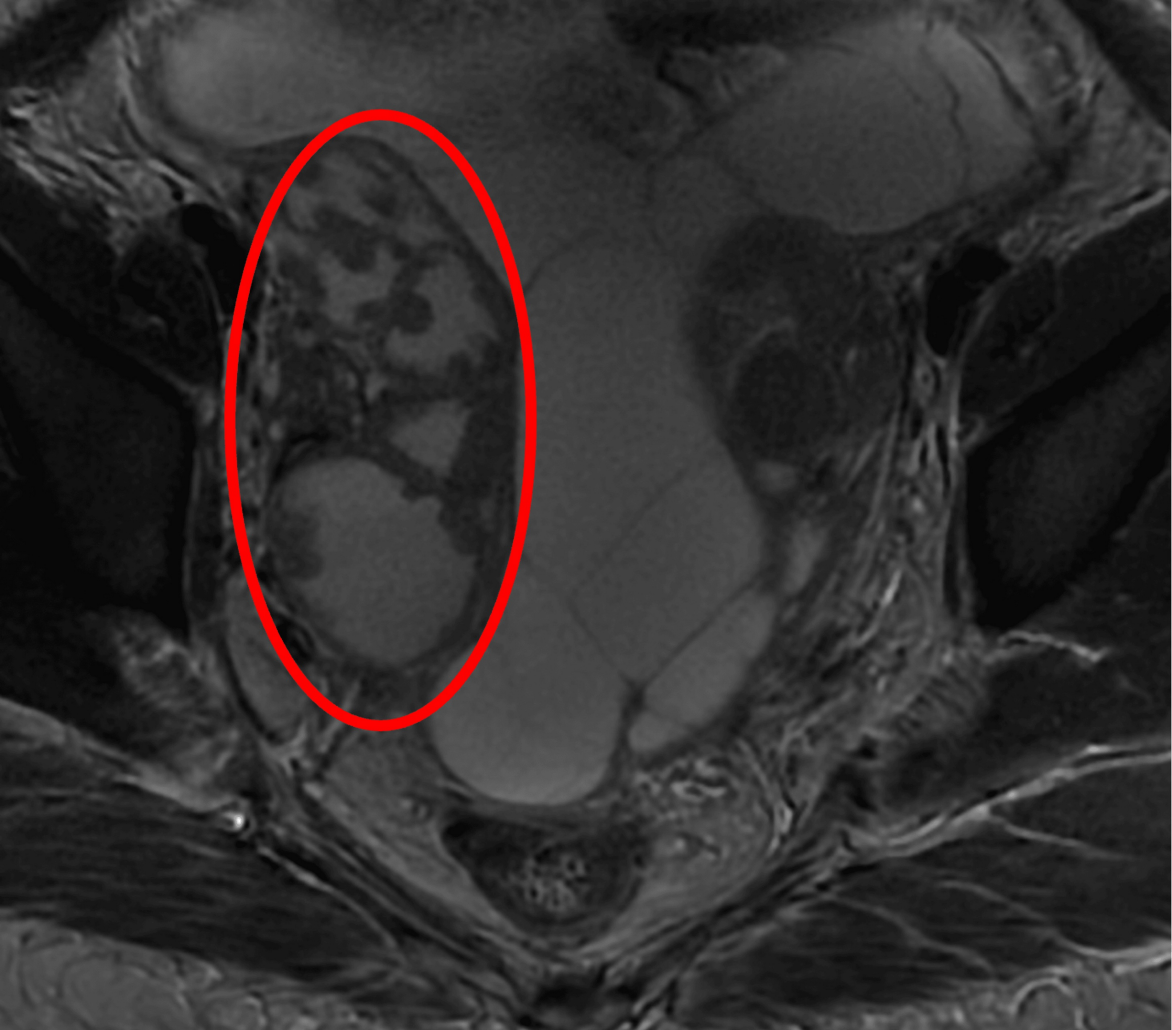
Axial T2-weighted pelvis MRI with contrast reveals a hydrosalpinx with a thick-walled fallopian tube showing marked enhancement and restricted diffusion, indicating pyosalpinx (circle)

To enhance visualization of the adnexa, a magnetic resonance imaging (MRI) of the pelvis was conducted. The MRI revealed that the pelvic masses, previously thought to be bulky ovaries, were actually bilateral hydrosalpinx, more pronounced on the right side. The findings were suspicious for pyosalpinx, associated with septated ascites and peritoneal thickening. Overall, the appearances suggested an inflammatory or infective etiology (Figure 2).

Initially, the patient was started empirically on antibiotics, including ceftriaxone and metronidazole, for peritonitis and bacterial vaginosis. Blood culture results showed *Staphylococcus epidermidis* and *Staphylococcus capitis*, which were the most likely contaminants.

A paracentesis was performed, yielding pale yellow translucent ascitic fluid for analysis. The results indicated a serum ascites albumin gradient (SAAG) of less than 1.1 g/dL and features suggestive of neutrophil-predominant peritonitis (Table 2). Xpert® MTB/RIF (Cepheid, Sunnyvale, California, United States) tested positive. Peritoneal fluid histopathology showed numerous mixed inflammatory cells and scattered reactive mesothelial cells with no evidence of malignancy. Acid-fast bacilli (AFB) smear and culture showed growth of pan-sensitive *M. tuberculosis* in four weeks.

**Table 2:** Peritoneal fluid analysis test results RBC: red blood cell; LDH: lactate dehydrogenase

Laboratory test	Patient Value	Reference Range
RBC (x10^6/L)	2,000	<1000
Nucleated cells (x10^6/L)	720	<250
Neutrophils (%)	53.0	
Macrophages (%)	20.0	
Albumin (g/L)	20	35-50
Protein (g/L)	53	66-87
Glucose (mmol/L)	1.3	7-10
LDH (IU/L)	1,234	160-320
pH	8.00	>7.5-8.0

Active pulmonary TB was ruled out after three AFB sputum smear samples, and the patient was discharged with anti-tuberculosis treatment for 8-12 months. She was deemed fit for travelling back to her home country, but was unfortunately lost to follow-up.

## Discussion

Female genital TB (FGTB), a type of EPTB, was first described by Morgagni in 1744 during an autopsy of a young woman who died from TB peritonitis. Genitourinary TB constitutes 27.1% of EPTB cases, with genital TB accounting for 9% of these. The exact incidence of FGTB is unclear due to underreporting, asymptomatic cases, nonspecific symptoms, and a lack of sensitive diagnostics. FGTB is often indolent, with patients rarely reporting abdominal pain, abnormal genital bleeding, or dyspareunia. Sometimes, clinical and radiological symptoms like ascites or abdominal distension may mimic malignant tumors, especially in older patients [3].

FGTB is an incidental finding in infertility investigations, primarily affecting the fallopian tubes and endometrium [5]. It spreads like other forms of EPTB and can also be sexually transmitted through an infected male partner's semen [3]. In developed countries, the average age for developing FGTB is 40 years, while in Asia, it occurs earlier (20-30 years) due to early marriage and childbearing. FGTB causes menstrual irregularities and infertility. Timely diagnosis and proper combination therapy can reduce damage and help prevent future infertility in affected women [3].

Diagnosing FGTB and peritoneal TB involves multiple diagnostic tools. The disease often remains silent and presents with vague symptoms, making diagnosis challenging. More than 90% of women with FGTB have involvement of the fallopian tubes, with both tubes usually affected. The infection can present as TB endosalpingitis, exosalpingitis, interstitial TB salpingitis, or salpingitis isthmica nodosa [6]. The acute phase of TB salpingitis is characterized by exudation, during which hydrosalpinx or pyosalpinx can develop, as seen in our patient. In contrast, the productive-adhesive type is typically observed during surgery, showing thickened nodular tubes that are tightly adhered to surrounding organs. Over time, calcification and fibrosis may occur as part of the healing process [6].

Diagnostic paracentesis should be performed in all patients with ascites in whom peritoneal TB is suspected. The ascitic fluid is typically straw-colored, although it can occasionally appear hemorrhagic. Signs indicative of peritoneal TB include an ascitic fluid protein level of ≥2.5 g/dL, SAAG below 1.1 g/dL in non-cirrhotic patients, an elevated adenosine deaminase level ranging from 36 U/L to 40 U/L, which has 100% sensitivity and 97% specificity, and a cell count between 500 and 1500 cells/mm³ accompanied by relative lymphocytic pleocytosis [7,8]. The case of the current patient is atypical because it involves neutrophilic ascites instead of the typical lymphocytic predominant count. This finding suggests either an underlying inflammatory process or an atypical presentation of TB. Upon reviewing the literature, neutrophil-predominant TB peritonitis was observed in individuals for whom TB complicates chronic peritoneal dialysis status or who have a history of peritonitis due to other causes [9,10].

Although a positive culture is considered the gold standard, ascitic fluid demonstrates low sensitivity of 45%-69% but high specificity of > 97% and can take weeks to yield results. Less than 5% of samples are smear-positive when stained using acid-fast or Ziehl-Neelsen stain [8]. 

Imaging aids diagnosis but lacks specificity. Ultrasound and CT help diagnose peritoneal TB, showing ascites, lymphadenopathy, thickening, nodules, septations, and adhesions [11]. They enable guided interventions like biopsies or fluid aspiration. Nonetheless, the gold standard remains laparoscopic visualization with biopsy. Laparoscopy is key for diagnosing peritoneal TB, allowing affected tissues to be visualized and biopsied. A notable feature is diffuse tan nodules on the peritoneum; turbid ascites and adhesions can also occur. Despite its importance, laparoscopic findings can mislead clinicians, as they may resemble abdominal malignancies like ovarian cancer [1]. A review of 402 patients showed laparoscopic examination had 93% sensitivity and 98% specificity for peritoneal TB diagnosis [12]. A Cochrane review indicated a pooled sensitivity of 50% for GeneXpert in diagnosing peritoneal fluid, which makes it not recommended for detecting abdominal TB [13]. However, it can be used as a supplementary diagnostic tool, as false-positive results are rare, approximately 2%, making a positive result reliable evidence of EPTB that can help guide final clinical decisions [14], as was the case with our patient. Thus, the best diagnostic method remains laparoscopic biopsies, although they are invasive and may have side effects.

The AF stain and culture require adequate samples, which are hard to obtain due to the paucibacillary nature of FGTB lesions. This necessitates histopathological analysis if there is a strong suspicion of malignancy. Histology typically reveals granulomas with giant multinucleated and epithelioid cells and caseous necrosis, though early TB may not show these features. Proper sampling during laparoscopic procedures is essential due to risks from adhesions. FGTB rarely shows macroscopic caseous necrosis, underscoring the need for careful sampling [5].

CA125 is a crucial tumor marker for diagnosing ovarian cancer. This glycoprotein is present in the cells of the uterine endometrium, and its serum levels increase in conditions such as ovarian malignancy, endometriosis, and pelvic inflammatory disease [15]. Furthermore, CA125 is expressed in cells lining the pleura, pericardium, and peritoneum, resulting in elevated serum levels in peritoneal TB, intestinal cancers, and postoperatively, making it non-specific [15]. Because of overlapping clinical features and high serum CA125 levels, TB ascites are often misidentified as malignancy [16]. This misdiagnosis is especially concerning in female patients with ascites, resulting in unnecessary invasive laparotomies. There is no established cutoff for serum CA125 levels to differentiate TB ascites from ovarian cancer. It may serve as an effective marker for diagnosing and monitoring patients with TB peritonitis [16]. Unfortunately, our patient had only one result; no serial tests were conducted due to loss of follow-up.

According to the Infectious Diseases Society of America guidelines, a six-month regimen is sufficient for managing peritoneal TB. The standard treatment generally involves two months of a four-drug regimen (rifampin, isoniazid, pyrazinamide, ethambutol) followed by four months of rifampin and isoniazid. Evidence regarding the use of adjunctive corticosteroids in TB peritonitis is limited and experts typically advise against their routine use [17].

## Conclusions

Diagnosing peritoneal TB and FGTB can be mistaken for ovarian cancer or peritoneal carcinoma due to overlapping clinical, laboratory, and imaging findings such as elevated CA125 levels, abdominal pain, ascites, and pelvic masses. Despite its rarity, it should be included as a differential diagnosis. Imaging modalities may assist in the diagnosis until further exploration, particularly diagnostic laparoscopy with biopsy, is required for suspected ovarian cancer and EPTB.
